# Editorial: Role of tissue-localized regulatory T cells in health and disease

**DOI:** 10.3389/fimmu.2025.1718891

**Published:** 2025-10-28

**Authors:** Bevin Smith, Diane Mathis, Ali A. Zarrin

**Affiliations:** ^1^ TRex Bio Inc., South San Francisco, CA, United States; ^2^ Department of Immunology, Harvard Medical School, Boston, MA, United States

**Keywords:** Treg, CD4+Foxp3+ regulatory T cells, inflammation, tissue immunology, autoimmunity

Regulatory T cells (Tregs) are indispensable for maintaining immunologic balance (1, 2). Far from being passive suppressors, tissue-localized Tregs emerge as dynamic players that integrate immunologic signals with local cues to shape repair, regeneration, and disease progression. This Research Topic brings together studies that dissect how Tregs show epigenetic and functional adaptations to their tissue environments and how these determine outcomes in autoimmunity, cancer, infection, and fibrosis.

The identity and stability of Tregs depend on Foxp3, which is maintained by a network of epigenetic and transcriptional regulators. In this Research Topic, Christensen et al. and Subramanyam et al. revealed how distinct coregulators safeguard this lineage. Loss of Sin3a, a chromatin-modifying cofactor of Hdac1/2, destabilizes Foxp3 and reduces Tregs, leading to impaired immune regulation. Deletion of the transcription factor CREB in Tregs reduces Foxp3 expression and polarizes to a Th2-like phenotype displaying co-expression of ST2, IL-10, and IL-13. The functionality of these Tregs is retained, and the enhanced production of IL-10 mediates a protective effect in a colitis model using T cell transfer. Together, these studies underscore the diverse ways in which coregulators affect Foxp3 stability and function and highlight the need to better understand how such pathways shape tissue-localized Tregs in humans.

Genetic background shapes Treg composition and function. Santamaria et al. identified a genomic region that may promote GITR^high^PD-1+ autoreactive Tregs in the NOD mouse model of type 1 diabetes. In contrast, Liu et al. sought to establish a causal relationship between genetic variants and disease outcome. By subsetting Tregs into specific fractions using combinations of surface markers, the authors found genetically predicted associations between the CD39+CD8+ regulatory cells in the co-occurrence of autoimmune disorders and certain lymphomas. Together, these studies illustrate how germline variation can shape Treg compartments, highlighting the need to integrate developmental and genetic perspectives to understand Treg biology.

The migration and retention of tissue Tregs depend on their repertoire of adhesion molecules along with chemokine receptors. Cheru et al. showed that, although tissue-localized Tregs share a core identity across organs, they are profoundly shaped by local microenvironmental cues, giving rise to heterogeneous phenotypes and functions. Cell-to-cell interactions and cell-independent influences within the tissue microenvironment contribute to the transcriptional profiles of Tregs. Xiang et al. expanded on this concept by highlighting how reciprocal interplay between these tissue-specific elements on both Tregs and effector T cells, the conflicting Yin and Yang, is critical in maintaining the immunological steady state, and how imbalances may be key drivers of disease. Together, these studies emphasize that tissue residency is not simply about localization; it is also about continuous dialogue with the surrounding niche.

The impact of Tregs on disease outcomes is profoundly shaped by context. For example, in neonatal sepsis, Sossou et al. reported that, although circulating Tregs decline overall, the fraction of activated Tregs rises, coinciding with reduced effector T cell responses and greater disease severity. In cancer, Pereira et al. highlighted how tumor-infiltrating Tregs influence both immunological and stromal compartments. Building on these findings, Zheng et al. reviewed co-signaling pathways in cancer and autoimmunity, pointing to opportunities for therapeutic targeting of Treg interactions. Together, these contributions underscore that Tregs are not inherently protective or pathogenic; rather, their effects are dictated by the tissue and disease context in which they operate.

Beyond immunological regulation, tissue-localized Tregs also participate in repair and remodeling under steady states and during acute inflammation. Wang et al. reviewed how Tregs regulate innate and adaptive immune responses and promote cardiac tissue repair in cardiovascular diseases. While shown to positively improve post-infarction tissue repair upon adoptive transfer, Tregs have also been reported to drive the aberrant proliferation of fibroblasts, leading to fibrosis. Zhang et al. expanded on this concept in multiple tissues by describing how Treg-derived mediators, such as amphiregulin, can either prevent or exacerbate fibrotic remodeling depending on the context. These dual roles highlight a central theme: tissue Tregs are potent orchestrators of regeneration; however, their reparative programs can tip into pathology when unchecked. 

## Conclusions

The articles in this Research Topic illustrate that tissue-localized Tregs are far more than static suppressors of immunity: they are dynamic, context-dependent players that integrate transcriptional, genetic, and environmental cues to shape outcomes across health and disease. From epigenetic mechanisms that safeguard Foxp3 stability to genetic influences that bias Treg differentiation to microenvironmental signals that diversify their tissue functions, each contribution underscores the flexibility of this lineage. These disease-focused studies further reveal the dual nature of Tregs, which are protective in some contexts and pathogenic in others, whether in infection, cancer, or fibrotic repair. Looking ahead, the field faces the challenge of translating these mechanistic insights into therapeutic strategies that can enhance or restrain Tregs in a tissue- and disease-specific manner. As the boundary between tolerance, immunity, and regeneration continues to blur, understanding the principles that govern tissue Treg biology will be essential for designing the next generation of effective immunotherapies.
